# Mobile Phone Apps for Low-Income Participants in a Public Health Nutrition Program for Women, Infants, and Children (WIC): Review and Analysis of Features

**DOI:** 10.2196/12261

**Published:** 2018-11-19

**Authors:** Summer J Weber, Daniela Dawson, Haley Greene, Pamela C Hull

**Affiliations:** 1 Division of Epidemiology Department of Medicine Vanderbilt University Medical Center Nashville, TN United States; 2 Leighton School of Nursing Marian University Nashville, TN United States; 3 Department of Epidemiology School of Public Health University of Alabama at Birmingham Birmingham, AL United States

**Keywords:** WIC, low-income, mobile phone, mHealth

## Abstract

**Background:**

Since 1972, the Special Supplemental Nutrition Program for Women, Infants, and Children (WIC) has been proven to improve the health of participating low-income women and children in the United States. Despite positive nutritional outcomes associated with WIC, the program needs updated tools to help future generations. Improving technology in federal nutrition programs is crucial for keeping nutrition resources accessible and easy for low-income families to use.

**Objective:**

This review aimed to analyze the main features of publicly available mobile phone apps for WIC participants.

**Methods:**

Keyword searches were performed in the app stores for the 2 most commonly used mobile phone operating systems between December 2017 and June 2018. Apps were included if they were relevant to WIC and excluded if the target users were not WIC participants. App features were reviewed and classified according to type and function. User reviews from the app stores were examined, including ratings and categorization of user review comments.

**Results:**

A total of 17 apps met selection criteria. Most apps (n=12) contained features that required verified access available only to WIC participants. Apps features were classified into categories: (1) shopping management (eg, finding and redeeming food benefits), (2) clinic appointment management (eg, appointment reminders and scheduling), (3) informational resources (eg, recipes, general food list, tips about how to use WIC, links to other resources), (4) WIC-required nutrition education modules, and (5) other user input. Positive user reviews indicated that apps with shopping management features were very useful.

**Conclusions:**

WIC apps are becoming increasingly prevalent, especially in states that have implemented electronic benefits transfer for WIC. This review offers new contributions to the literature and practice, as practitioners, software developers, and health researchers seek to improve and expand technology in the program.

## Introduction

The Special Supplemental Nutrition Program for Women, Infants, and Children (WIC) provides nutritious foods, nutrition education, and health care referrals to low-income pregnant, postpartum, and breastfeeding women and children from birth until their 5th birthday in the United States. Since its inception in 1972, WIC has been associated with improving health outcomes of participants, including but not limited to positive impacts on nutrient and dietary intakes [1,2], childhood obesity rates [3], household food security [4], immunization rates [5], and birth outcomes [6]. As one of the only federal nutrition programs designed to monitor the health of participants, WIC has been a fundamental contributor toward research surrounding maternal and child health for decades and has been proven to be one of the most successful and cost-effective nutrition intervention programs in the United States [7].

The benefits of WIC participation are clear; however, participation in WIC has declined since 2010 [8], and the program is in need of updated tools to help future generations. Barriers to participating in WIC exist, especially when shopping for WIC foods at the store. Participants often face difficulty finding the correct foods on their family’s WIC food list and problems with redeeming benefits during checkout [9,10]. Clinic appointment wait times and scheduling issues [9,11] have also been reported as program barriers. To receive food benefits and remain enrolled, WIC participants are required to complete periodic nutrition education. Some participants face difficulties attending these classes in person [9]. Many WIC agencies have attempted to reduce this burden by allowing participants to complete some nutrition education requirements online. As of 2016, 34 states and 5 intertribal councils used some form of online nutrition education [12].

Electronic benefits transfer (EBT) for WIC is currently being implemented in several states and is mandated for all states by 2020 [13]. EBT is a mechanism for using WIC benefits like a debit card to make WIC transactions less cumbersome for participants and vendors alike [14]. By eliminating the need for paper vouchers, participants no longer need to redeem all benefits on a voucher in a single transaction, wait for the cashier to verify each food, and sign each voucher like a check. This paper voucher process has been known to cause holdups at checkout, which adds stigma and embarrassment [11].

Although EBT has been found to improve redemption and ease at checkout [15], it has not been associated with an increase in enrollment [16] and cannot alleviate all the problems associated with shopping for and choosing the correct WIC foods. Mobile phone apps for WIC, therefore, are becoming increasingly prevalent and necessary to assist participants with the WIC shopping experience [17,18]. Several WIC apps have been designed to help participants navigate the program and its benefits; however, these apps offer a variety of features, and few have been assessed in the scientific literature [17,18]. As part of our team’s efforts to develop a new app for the Tennessee WIC program called Children Eating Well (CHEW), we conducted a review of existing WIC apps. Specifically, this review aimed to identify publicly available apps for WIC participants, analyze their main features, and examine user review ratings and comments. This review will be useful for practitioners, software developers, and researchers to inform them about existing tools for WIC and guide planning for the incorporation of new technologies to improve the program.

## Methods

Keyword searches were performed between December 2017 and June 2018 in the largest online stores for the two most commonly used operating systems: the App Store for Apple iOS devices and Google Play for Android devices. Keywords used in the search were my WIC, WIC, and WIC app. The inclusion criteria for apps was being related in some capacity to the WIC program. The exclusion criteria were apps that were not targeted for use to program participants (eg, designed for vendors). Two coauthors screened the apps for eligibility.

The range of number of installations was documented for apps found in Google Play. The App Store, however, does not list the number of installations. The app rating and number of reviews were documented for each app for each store, if available. Each store displays a rating out of 5 possible points. The App Store does not provide a user rating if fewer than 5 users have submitted a review. The states where each app was available for use was also documented.

To review the features of the included apps, publicly available information was gathered from the App Store and Google Play store via preview images, descriptions, user ratings, and review comments. In addition, each app was installed on both iOS and Android devices to view features when possible. Two coauthors reviewed the apps and extracted data, and any differences were resolved through discussion with a third coauthor. Most WIC apps required user verification as an enrolled participant in the respective state to fully access the features. Therefore, it was not possible to test usability of the apps.

App features were then organized into major categories. To our knowledge, this is the first review of WIC-related mobile phone apps and features. Determining the major categories, therefore, was an inductive process supported by the authors’ familiarity and experience with the WIC program at the participant, clinic, vendor, and program administration levels.

## Results

### Search Results

**Figure 1:**
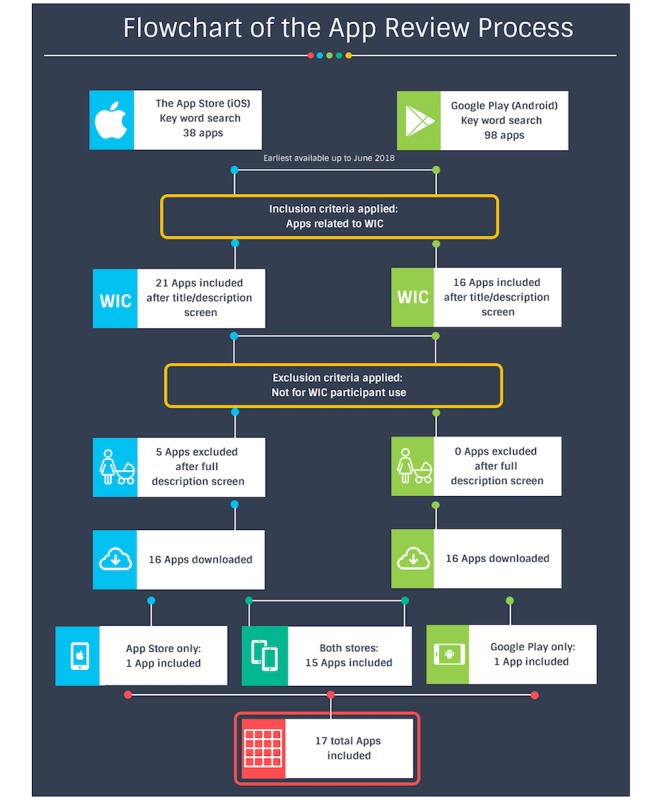
Flowchart of the app review process.

A total of 38 apps were found from the keyword search within the App Store, 21 of which were related to WIC (see Figure 1). Five WIC apps were excluded from the App Store results because they were not targeted for users who are WIC participants; instead, they targeted WIC vendors and agents to view and manage eligible products. In the Google Play store, 98 apps were found from the keyword search, 16 of which were related to WIC. Fifteen WIC apps were duplicates in both online app stores, while 1 app (SAC WIC) was available for iOS only and 1 app (AZ WIC Clinic Search) was available for Android only. This resulted in a final total of 17 apps targeted toward WIC participants included in the review. Two apps, SAC WIC and AZ WIC Clinic Search, however, were removed from the online stores during the time of this review.

### States and User Ratings

Although WIC is a federal program used nationwide, it is administered at the state and local level. Therefore, WIC apps were designed for specified states, territories, or tribal nations. Currently 30 states and Washington DC, 3 US territories (American Samoa, Guam, and the Commonwealth of Northern Mariana Islands), and 3 tribal nations have WIC-related apps. Several states (n=6) had more than one WIC-related app; however, these did not overlap in content or features (eg, nutrition education vs shopping). All apps were free to download; however, most apps (12/17, 70%) required the user to register with the app to verify WIC participation so the user could view and use features specific to his or her own WIC ID number (eg, food benefit balance or appointment times). Some apps (5/17, 29%) contained features that could be readily accessed by any public user, regardless of WIC enrollment or residence in the state for which it was developed. User verification requirements for each app are displayed in Table 1.

WIC apps and their corresponding states, ratings, number of ratings, and range of installations (for Android only) are also displayed in Table 1. A total of 41% (7/17) of WIC apps fell into the 10,000+ downloads category in Google Play. The most frequently installed (500,000+ downloads in Google Play) WIC-related app was WIC Shopper. WIC Shopper’s relatively high install rate may be due to the fact that it was used by 18 states and agencies, all of which have begun to implement EBT for WIC. Only 2 other apps were being used by multiple states and agencies (WIC Smart and EzWIC). User ratings varied widely for both iOS and Android apps, and many were not available due to a lack of ratings. However, for those that had available data, Maryland WIC received the lowest rating in both stores (3.0 and 2.2, respectively).

Most apps received some negative reviews. According to user review comments in both app stores, many of the low ratings were due to the app crashing or freezing or an inability to log in due to system maintenance. User review commenters who gave low ratings also mentioned experiencing issues with registering their accounts, password reset, internet connectivity issues inside the grocery store, or using the app in a state for which the app was not designed to work. App developer responses to these negative reviews typically indicated they were working on resolving these issues or that the user needed to update their app for bug fixes and performance enhancements. For example, one user pointed out a challenge with the log-in process being too cumbersome and time consuming.

My only complaint is that the app won’t keep the login information saved. I keep having to type all the card info in whenever I want to use it. Not a terrible thing, just really inconvenient when I’m balancing a crying baby and want to take a quick look while grocery shopping.

Wisconsin MyWIC (iOS version) user review, 4 out of 5 stars

### Women, Infants, and Children Program App Features

App features fell into 5 main categories: shopping management features, clinic appointment management features, informational resources, WIC-required nutrition education modules, and other user input. Specific feature types under these main categories varied across the apps and are summarized in Multimedia Appendix 1.

#### Shopping Management Features

Most of the WIC apps requiring user verification (11/12, 92%) offered features to assist the WIC participant with real-time shopping for WIC foods in the grocery store using EBT. These shopping management features included real-time food benefit balance checking (10/17, 59%), barcode scanning (10/17, 59%) and manual PLU/UPC (price look-up and universal product code) entry (3/17, 18%) to check if an item was WIC eligible, benefit expiration reminders (9/17, 53%), future benefit viewing (5/17, 29%), and transaction dispute functions for if the user disagreed or did not understand why an item could not be redeemed with WIC (1/17, 6%). Seven apps also included shopping management features that allowed participants to view and navigate to the locations of WIC-approved vendors.

This is a great app. I like how everything is going paperless, like I do not have to keep the balance receipts. I can check on the app and scan the items to see if they are approved [and] also find stores that take WIC near me.

Wisconsin MyWIC (Android version) user, 5 out of 5 stars

Negative user reviews regarding shopping management features included comments about an app’s barcode scanner not working or not correctly determining WIC items. According to positive user reviews, benefit balance checking and bar code scanning features were highly valuable to users trying to complete their WIC shopping.

It’s time saving. You don’t have to stay for long at the cashier anymore!

EzWIC (Android version) user, 5 out of 5 stars

I love this app! It makes using WIC so much easier. Instead of getting to the register and realizing I picked the wrong thing, I can scan it right there and check it. It lets me know exactly how much I have left of each item. And I can look up a list of what I can buy. It’s so much nicer than having to carry around the bright orange folder and stop in the middle of the store and search and search to see if I was buying the right thing. I love that I can see what I have left and that it updates immediately...

WIC Shopper (Android version) user, 5 out of 5 stars

Everything about this app is awesome! I like the fact that I don’t have to carry paper around. Lets you be discreet about your business.

Wisconsin MyWIC (Android version) user, 5 out of 5 stars

Very helpful being a busy mom with young children to just check the app to see what I have left.

Bnft (Android version) user, 5 out of 5 stars

Only one app, Bnft, allowed users to not only manage their WIC benefits but also their Supplemental Nutrition Assistance Program (SNAP, formerly known as Food Stamps) benefits.

Apps with features that enabled benefit balance viewing accessed this information by syncing with the EBT vendor contracted through each state. If EBT access was not provided to the app developer in specific states, users could not access this feature, even if it existed in other states.

I like the version of this app for other states but not for Texas. I can’t see my benefits; I have to upload a photo of my WIC receipt... [WIC Shopper (Android version) user review, 2 out of 5 stars] Reply from JPMA, Inc: Unfortunately, [that] is a limitation of Texas WIC and not the app. If they could support card registration, we surely would add it.

**Table 1:** Mobile phone apps for Women, Infants, and Children programs available for public download.

App name (developer)	State or WIC^a^ agency	Range of installations (Android only)	App rating out of 5 (Android, iOS)	Number of ratings (Android, iOS)	Requires user verification to access features
Bnft (Soltran, Inc)^b^	North Carolina	10,000+	4.4, 3.6	18, 9	x
EzWIC (Arizona Department of Health Services)^b^	Arizona, American Samoa, CNMI^c^, Guam, Navajo Nation	50,000+	4.0, 4.8	69, 20	x
Indiana WIC (Indiana Office of Technology)	Indiana	10,000+	3.4, 4.8	112, 19	x
Maryland WIC (3 Sigma Software Inc)	Maryland	10,000+	3.0, 2.3	70, 29	x
My Minnesota WIC App (Minnesota Development Team)^d^	Minnesota	10,000+	4.2, —	29, —	x
My Oklahoma WIC (My WIC Development Team)	Oklahoma	1000+	5.0, —	1, —	x
MyWIC (Mobile Benefits Inc)	Chickasaw Nation	100+	Not available; beta version	Not available; beta version	x
WIC Connect (State of Michigan)	Michigan	10,000+	3.9, —	11, —	x
WIC Shopper (JPMA)	Connecticut, Colorado, Florida, Iowa, ITC^e^ Arizona, Kansas, Kentucky, Massechusetts, Montana, Nevada, New Mexico, Oregon, Texas, Vermont, West Virginia, Washington DC, Wyoming	500,000+	3.9, 4.6	2966, 442	x
WICSmart^f^ (JPMA)	Arkansas, Choctaw Nation, Connecticut, ITC Arizona, Massechusetts, Montana, Rhode Island, West Virginia, Pennsylvania, Washington DC	10,000+	3.3, —	26, —	x
WIC2Go (3 Sigma Software Inc)	New York	500+	1.0, 4.7	1, 7	x
Wisconsin MyWIC (Wisconsin Department of Health Services)	Wisconsin	10,000+	4.7, 2.4	97, 19	x
Alabama WIC (OCV, LLC)	Alabama	5000+	4.6, —	19, —	
Arizona WIC Clinic Search (Arizona Department of Health Services)	Arizona	No longer publicly available	No longer publicly available	No longer publicly available	
SAC WIC (Social Interest Solutions)	Sacramento County, California	No longer publicly available	No longer publicly available	No longer publicly available	
WIC Food Shopping Guide (WYWICAPP)	Wyoming	1000+	4.3, —	6, —	
WIC San Diego (SDSU WIC)	San Diego County, California	500+	4.5, —	6, —	

^a^WIC: Special Supplemental Nutrition Program for Women, Infants, and Children.

^b^Contains shopping features only.

^c^Commonwealth of the Northern Mariana Islands.

^d^Requires user verification for appointment reminder features only.

^e^Intertribal Council.

^f^Contains WIC-required nutrition education features only.

**Figure 2:**
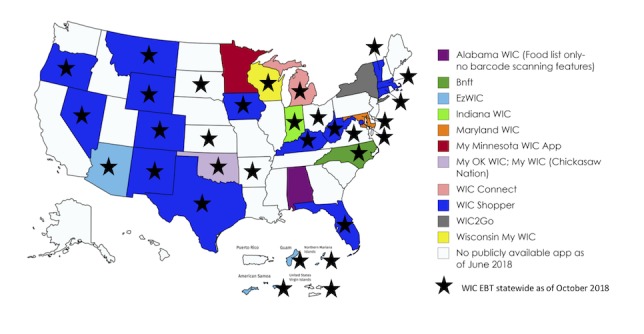
States and territories with shopping apps and electronic benefits transfer for Women, Infants, and Children program participants.

As of June 2018, 24 states had not yet fully implemented EBT for WIC [13] and were still using paper-based vouchers; therefore, apps with shopping management features were not available to participants in most non-EBT states. Minnesota, however, is the only one of these paper-based voucher–based states with a shopping app available for public download with features including barcode scanning and PLU/UPC entry to determine WIC eligible items but without the ability to verify if it is available on a specific user’s balance. Like the apps from other states that do not share EBT information with the app developer, My Minnesota WIC App is unable to display participants’ own real-time benefit balance since the state does not manage benefits electronically. Figure 2 shows available WIC apps with shopping management features in the United States.

#### Clinic Appointment Management Features

Most WIC apps requiring user verification (9/12, 75%) also included at least one feature to help participants manage and navigate their WIC clinic visits. Eight apps had features that allowed participant users to view their future appointment date and provided a list of required documents needed for the upcoming appointment. In 3 of these apps, users could also receive notifications for appointment reminders based on information that is synced with the clinic. Alabama WIC also had an appointment reminder feature. However, this app requires no user verification, and the user must manually enter the information about their visit to set up a reminder, similar to a standard reminder feature included on mobile phones; therefore, this feature was not counted as a clinic management feature for the app. User review comments for several apps indicated desire for an app feature that would allow appointment scheduling and viewing.

I like the app but I would give 5 stars if you were [...] able to change your appointment thru the app...

Indiana WIC (Android version) user, 3 out of 5 stars

Although no app in this review included a feature to schedule or reschedule an appointment, Michigan’s WIC Connect had a feature that allowed participants to submit a request to the WIC office for a specific date and time for their next appointment, and the WIC office would contact them to schedule the appointment. Michigan’s app also allowed participants to update their contact information (address, phone number, email address), which could potentially assist WIC staff in contacting the participant for new or missed appointments.

Several apps (5/17, 29%) provided clinic locations with contact information and hours. AZ WIC Clinic Search was an app that solely provided clinic location features and clinic photos for potential and current participants; however, during the time of this review, EZ WIC Arizona added both vendor and clinic location features to its app, which eliminated the need for Arizona to have 2 separate apps. Thus, the AZ WIC Clinic Search app was removed from the app store. SAC WIC and WIC San Diego did not offer clinic management features; however, these apps did contain Web links to the California WIC Web portal (wic.ca.gov), which allowed verified WIC participants to view their upcoming appointments and voucher balance. User reviews did not provide positive or negative feedback about the clinic management features.

#### Informational Resource Features

General information resources were provided in 4 of the 5 WIC apps that did not require user verification and three-fourths (8/12, 67%) of the apps requiring user verification. The most common (10/17, 59%) was enabling participants to view a general WIC food list or simply providing a link to a webpage that contained their state’s general WIC food list. Other informational resource features included external links to videos or social media (6/17, 35%), breastfeeding resources (5/17, 29%), WIC eligibility information (3/17, 18%), community resources (2/17, 12%), and nonrequired nutrition education (2/17, 12%). Examples of nonrequired nutrition education included WIC tips (eg, tips on how to use WIC efficiently, general nutrition, and food preparation) and recipes with WIC foods. User reviews did not provide positive or negative feedback about these informational resource features. Users of apps that did not contain these features, however, did state that they would enjoy them.

I like the app but I would give 5 stars if you were able to add [...] a nutrition/ healthy eating section with recipes and ideas to help people learn to eat better with healthy choices not just the WIC-approved foods but any healthy option.

Indiana WIC (Android version) user, 3 out of 5 stars

#### Required Nutrition Education Modules as App Features

Alabama WIC, Indiana WIC, WIC Connect, SAC WIC, and WIC San Diego apps contained Web links to portals where participants could log in and complete required nutrition education modules on their phones instead of going in person to the WIC clinic. Only one app, WIC Smart, was designed to allow participants to complete these modules within the app itself and enabled the corresponding WIC state or agency to tailor these modules. According to comments in user reviews, apps that included features or links to WIC nutrition education requirements were perceived as useful to participants who could access them.

Needs updates its locked [...] please fix it asap lots of people use this to save time and gas money please help.

WICSmart (Android version) user, 3 out of 5 stars

#### Other User Input Features

SAC WIC and WIC San Diego were the only 2 that contained features that allowed any (nonverified) user to submit feedback about the app to the WIC program through the app. Wyoming’s WIC Food Shopping Guide was the only app that contained input features that allowed any (nonverified) user to complete an app usefulness poll or report WIC fraud.

## Discussion

### Principal Findings

During the time of this review, 17 mobile phone apps for WIC participants existed in 37 states, US territories, and tribal nations. WIC apps that assisted participants with real-time shopping management received the most positive user ratings and reviews. The most common features of these apps included WIC benefit balance checking and barcode scanning features. These advanced features were not available on apps that required no user verification. Two of these apps were removed from the app stores during the time of this review. Although WIC apps varied in user ratings and features, the ongoing development of technology to help low-income families navigate government nutrition assistance programs is promising and continues to grow as EBT for WIC becomes implemented throughout the United States.

Despite the recent expansion of EBT in WIC, EBT technology is not new in federal nutrition programming. SNAP first started implementing EBT for its participants and vendors in 1984, and by 2004, EBT for SNAP was nationwide [19]. The transition from paper to EBT in WIC, however, has been more challenging due to the complex nature of WIC food restrictions. WIC-authorized foods must meet specific nutrition criteria, whereas foods purchased with SNAP do not need to meet any nutrition-related guidelines [20]. Each of the WIC participant eligibility categories is issued a unique food package, or “food prescription,” that varies in the specific type and amount of allowed food items based on age and special dietary needs, whereas allotted SNAP benefits vary only by dollar amount based on household size and income level of the recipients.

As families continue to face stigma in using government nutrition assistance programs [21,22], updated technology such as EBT and mobile phone apps for SNAP and WIC could potentially make access to program benefits more discreet and acceptable. Despite the need for technological updates within these programs [23], barriers to innovation exist due to the complex nature of contractual agreements between the private and public sectors. WIC app developers who wish to give users the ability to view their benefit balance in real time must gain access to data from the EBT vendor contracted though the WIC state agency for which the app is designed to work. This process requires permission to access these Web-based services and can potentially be a hurdle for app developers, as was found for the app in Texas.

Web-based resources to help participants navigate WIC are not new to the program; however, existence of these resources is variable depending on the state agency. These tools include state-based webpages by which users can learn about WIC, check benefit balances, determine eligibility, locate clinics/vendors [24], and access nutrition education modules that can be completed online (rather than in person at the clinic) [25,26]. A recent study assessing preferences for Web-based technology apps within WIC has shown that most participants had access to the internet and own cell phones [27]. The study’s findings aligned with the current review in that most WIC participants reported that it would be very useful to access EBT balance (6678/8144, 82%), UPC scanning (5782/8144,71%), appointment scheduling (5212/8144,64%), recipes and cooking demos (5130/8144,63%), store locations (5049/8144,62%), and nutrition education (4804/8144,59%) through online technology [27].

Although low-income families’ access to technology continues to pose a limitation for Web-based health interventions, the digital divide is not the same as it was 10 or even 5 years ago due to the surge of mobile phone access and app technology [28]. Many low-income families do have internet access but are underconnected, with mobile-only access [29]. According to Pew Research 2018 data, over 90% of 18- to 49-year-olds and two-thirds of all low-income adults owned a mobile phone. Only 45% of the lowest income category (<$30,000) had broadband internet access at home, yet low-income adults were the most likely to report only accessing the internet via a mobile phone (31%) [30]. This helps to highlight the need to provide mobile-based apps and mobile-friendly websites for the WIC population since they may not have a computer with internet at home. As health-related resources continue to move online [29], Healthy People 2020 has outlined objectives to improve internet access [31]. Programmatic technology developments through mobile phone apps are, therefore, increasingly important for these families trying to use public services for which they qualify.

### Limitations

This review includes only those apps for WIC that were publicly available as of June 2018 in app stores for Android and iOS. As EBT for WIC continues to roll out nationwide, more state agencies are projected to either develop or adopt existing apps for participant use. App ratings cannot reflect comprehensive preferences of all users since there is no requirement to rate the apps that are downloaded. Publicly displayed reviews could potentially reflect a high negativity bias, especially for newer apps that are still working out bug fixes. Since users can rate any app they download, negative bias may also have influenced ratings by users who tried to use an app in a nondesignated state. Finally, since most apps required WIC user verification to gain full access to the app functionality, it was not possible to test usability of the apps directly for this review.

### Conclusions

Mobile phone apps for families using federal nutrition programs are becoming increasingly prevalent, especially in states that have implemented EBT for WIC. Based on user reviews of the included WIC apps, developers of future app versions may consider including shopping management features that were mentioned as especially useful (eg, benefit balance viewing and barcode scanning) and expanding on clinic management features (eg, appointment scheduling) and nutrition-related informational features (eg, healthy recipe demos) that were suggested by users. Collaboration between WIC state agencies, contracted private sector EBT vendors, app developers, and researchers is necessary to create and evaluate apps that can help low-income families with children access healthy foods and nutrition services.

## Appendix

Multimedia Appendix 1 Classification of features in mobile phone apps for Women, Infants, and Children program participants.

## Abbreviations

CHEW: Children Eating Well
EBT: electronic benefits transfer
PLU: price look-up
SNAP: Supplemental Nutrition Assistance Program
UPC: universal product code
WIC: Special Supplemental Nutrition Program for Women, Infants, and Children
