# Supplementary figures and images for: Mobile Phone Apps for Low-Income Participants in a Public Health Nutrition Program for Women, Infants, and Children (WIC): Review and Analysis of Features
Source: JMIR Mhealth Uhealth. 2018 Nov 19;6(11):e12261. doi: 10.2196/12261 (PMC6277824; doi:10.2196/12261)

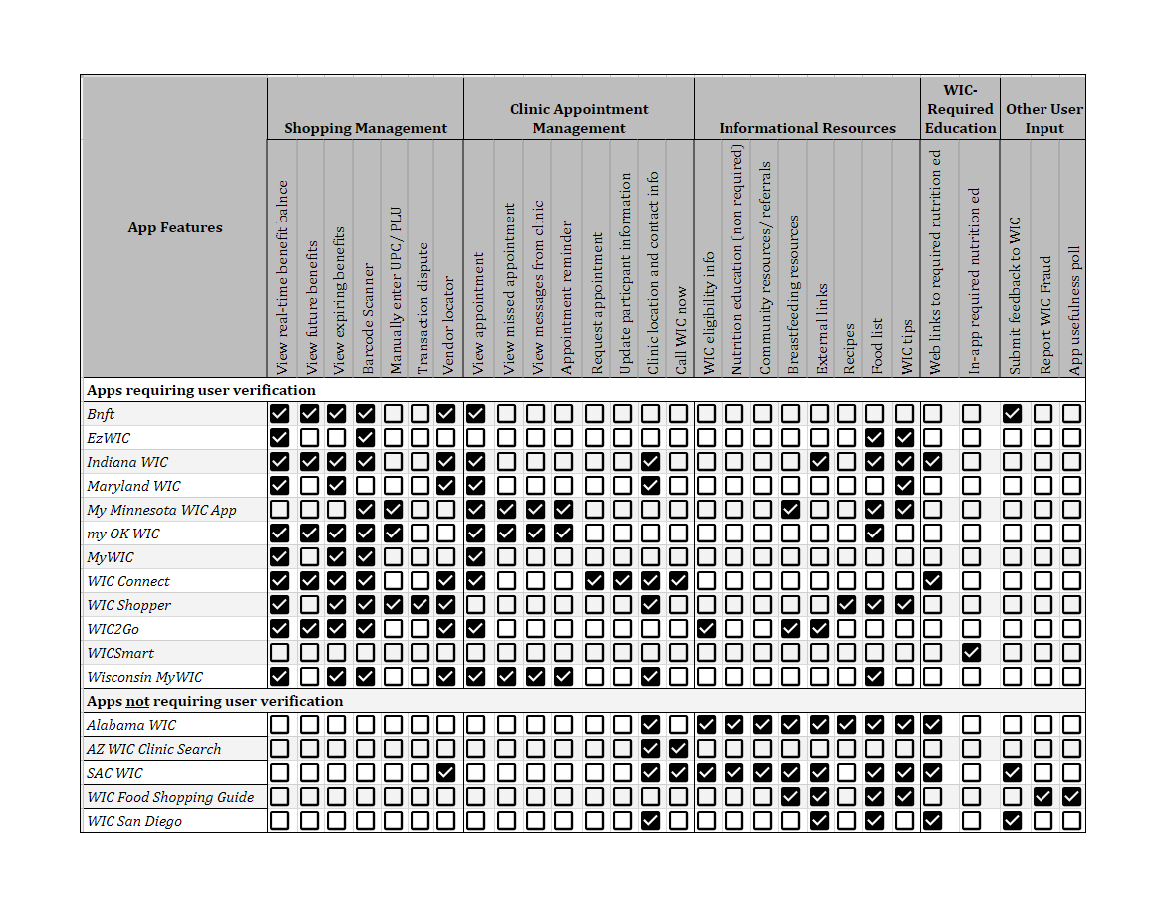

Supplement: Multimedia Appendix 1 [file mhealth_v6i11e12261_app1.png]
